# Determining the performance of a temperature sensor embedded into a mouthguard

**DOI:** 10.1038/s41405-022-00114-8

**Published:** 2022-08-01

**Authors:** Leonardo de Almeida e Bueno, William Milnthorpe, Jeroen H. M. Bergmann

**Affiliations:** Natural Interaction Lab (NIL), Department of Engineering Science, University of Oxford, Thom Building, Parks Road, Oxford, OX1 3PJ UK

**Keywords:** Dental equipment, Prosthetic dentistry

## Abstract

**Objective:**

This study aimed to determine the steady-state errors of oral-based temperature sensors, that are embedded in mouthguards, using a robust assessment process.

**Materials and methods:**

Four electronic boards with temperature sensors were encapsulated in mouthguards made from ethylene-vinyl acetate (EVA). The error and time to reach steady-state temperature were determined using a thermostatic water bath during three different conditions (34, 38.5 and 43 °C). Subsequently, a case study of one volunteer wearing the instrumented mouthguard is presented.

**Results:**

The water bath tests showed that a mean absolute error of 0.2 °C was reached after a maximum of 690 s across all test conditions. The case study yielded an absolute error was 0.2 °C after 1110 s.

**Conclusion:**

These results show that an instrumented mouthguard with temperature sensing capabilities can yield a consistent steady-state error that is close to the clinical requirements across a range of temperatures. However, the time it takes to reach steady-state temperature needs to be considered for these systems to correctly interpret the outcomes.

## Introduction

Monitoring intra-oral temperature is of clinical importance, as it is a key marker to assess patient status in both dentistry and medicine [1–4]. Sublingual temperature is related to core temperature and it can capture changes in body temperature which could signal the onset of infections, indicating a reaction to medications, or supports the monitoring of other disease-specific symptoms [5–7]. In dentistry, the intra-oral temperature can be a diagnostic tool to detect periodontal diseases, tooth erosion or decay [3, 8]. The intra-oral temperature has also been used to measure patient compliance by logging the time that oral appliances are worn [9–12]. In all these practices, obtaining an accurate temperature reading is essential.

Body temperature can be measured using a range of technologies that can meet a specific set of requirements consisting of e.g., a certain level of accuracy, specific device dimensions, or quickness of response. Typical methods for measuring intra-oral temperature include electronic sensor devices and single-use, heat-sensitive, chemical-strip temperature devices. Among the temperature sensors technologies used, thermocouples are often applied due to their wide temperature range, low cost and quick thermal response [7, 13]. Other technologies, such as resistance temperature detectors and semiconductor-based integrated circuits, also have been used for intra-oral temperature measurement, but are less common [13].

Studies on the accuracy of intra-oral temperature devices frequently focus on (1) the reproducibility of sensor’s readings over time in a specific temperature setting, (2) the accuracy of the sensor before it is encapsulated into the final product, or (3) the accuracy when measuring temperature in human subjects compared to a reference thermometer. However, when employing an intra-oral sensor, the circuit design, sensor technology, sensor placement, range of temperatures and even encapsulation method can all affect the measurement error. Ideally, tests should explore the performance of the encapsulated sensor by determining the error across a relevant range of temperatures. The mouth presents an environment with wide-ranging temperature variations, which must be properly measured for correct interpretation. For this reason, a clinical thermometer must be tested throughout the full range of interest for a specific application. In addition to determining the error for a given temperature range, one should also consider the time it takes for a sensor to reach temperature equilibrium or steady-state.

To our knowledge, no studies thus far have presented a “pre-clinical” simulation to evaluate both error and time components of wearable temperature monitors that have been embedded into a mouthguard. Thus, in this study, the error obtained against a gold reference and the time it takes to reach equilibrium across three different temperatures are investigated in a controlled lab-bench experiment. This experiment, based on ISO standards for clinical thermometers for body temperature measurement, simulated a set of clinically relevant temperatures using a thermostatic fluid bath, standard practice for thermometers calibrations [14]. The sensors are also tested in one human volunteer to preliminary explore the generalizability of the errors obtained from the lab bench. This case study provides data on a true intra-oral temperature monitoring condition.

## Materials and methods

### Instrumentation

**Fig. 1:**
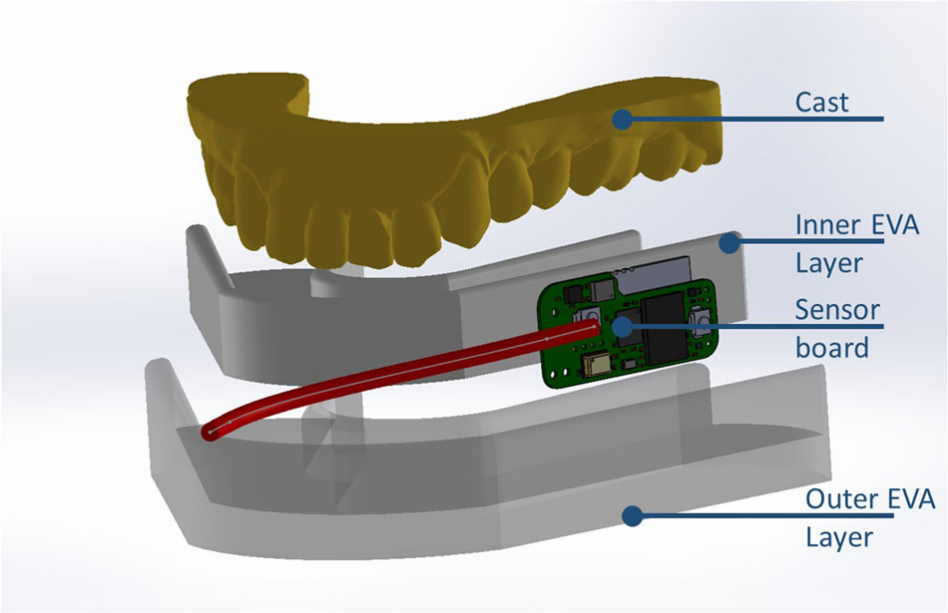
Schematic of the mouthguard assembly. A single 1.20 mm layer of EVA was thermoformed on the working cast. A wired custom-designed sensor board was placed between the first and second layer of EVA. The (red) wires were positioned to exit the mouth from the front.

**Fig. 2:**
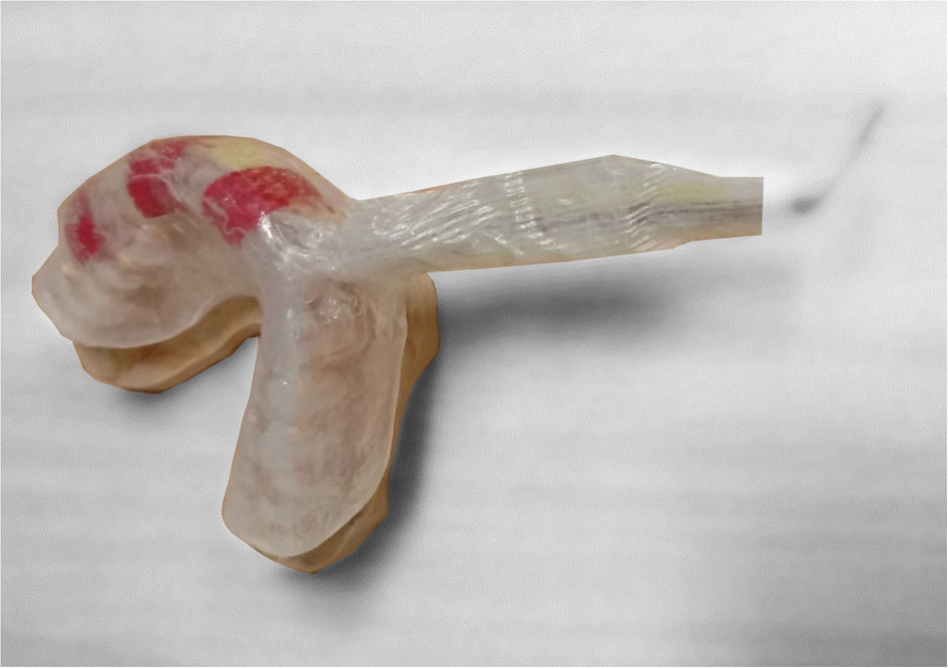
An EVA encased mouthguard situated on top of the cast with a data acquisition system embedded. A cable is used to connect it with the MAX30208 Evaluation System. Red electric tape is used to keep the wires in place during the forming process, while a yellow pH sensitive comparator paper strip is positioned along the wires to detect potential water ingression.

The oral monitoring device, based on the device used in ref. [15], consisted of a flexible design that continuously measures several intra-oral signals (including temperature) at the same time. It was vacuum formed using ethylene-vinyl acetate (EVA). A maxillary vinyl polysiloxane impression (R&S Turboflex) was taken in disposable perforated plastic impression (Medibase) and cast in vacuum mixed Type 4 dental stone (Singletypo4). A single 1.20 mm layer of EVA (Pro-form) was then applied on the working cast with a pressure-molding machine (Kezham XG-E01). The applied sheet was trimmed leaving a 2–3 mm margin along the gingival margins of the teeth and the posterior borders. A custom-designed data acquisition system containing an ARM Cortex-M4 microprocessor (STM32L476JGY, STMicroelectronics, Switzerland) capable of running at up to 80 MHz, a Bluetooth 4.2 network processor (BlueNRG-MS, STMicroelectronics, Switzerland), a flash memory (S25FS512SDSBHV213, Cypress Semiconductor, USA) and a digital temperature sensor (MAX30208, Maxim Integrated, USA), with ±0.1 °C manufacturer accuracy from +30 to +50 °C, were secured on the first layer of EVA using an appropriate adhesive (Loctite). A schematic overview of the design is provided in Fig. 1. For this study, the data acquisition board was connected to the MAX30208 Evaluation System to standardize the data collection using the MAX30208EVKitSetupV100 PC GUI program. The sensor board was placed at the buccal side of the upper first molar to avoid as much as possible intra-oral temperature variation, due to the inhalation related airflow [16, 17]. Since the aim is to assess sensor accuracy in a lab environment, the Bluetooth communication was not used. The device was tethered ensuring robust data collection throughout all experimental conditions. The wires from the measuring components (total length ~25 cm) were positioned to exit the mouth from the front, to minimize occlusal interferences. Red electric tape was used to keep the wires in place during the forming process (Fig. 2). A pH sensitive comparator paper strip was positioned along the wires to detect any potential water ingression. A second layer of EVA was applied once the board was in place, which then bonded to the first layer. Excess EVA was trimmed according to the outline of the previously applied layer. The edges were smoothed and rounded with a heated wax knife to improve the comfort when being worn intra-orally (Fig. 1).

### In vitro testing

**Fig. 3:**
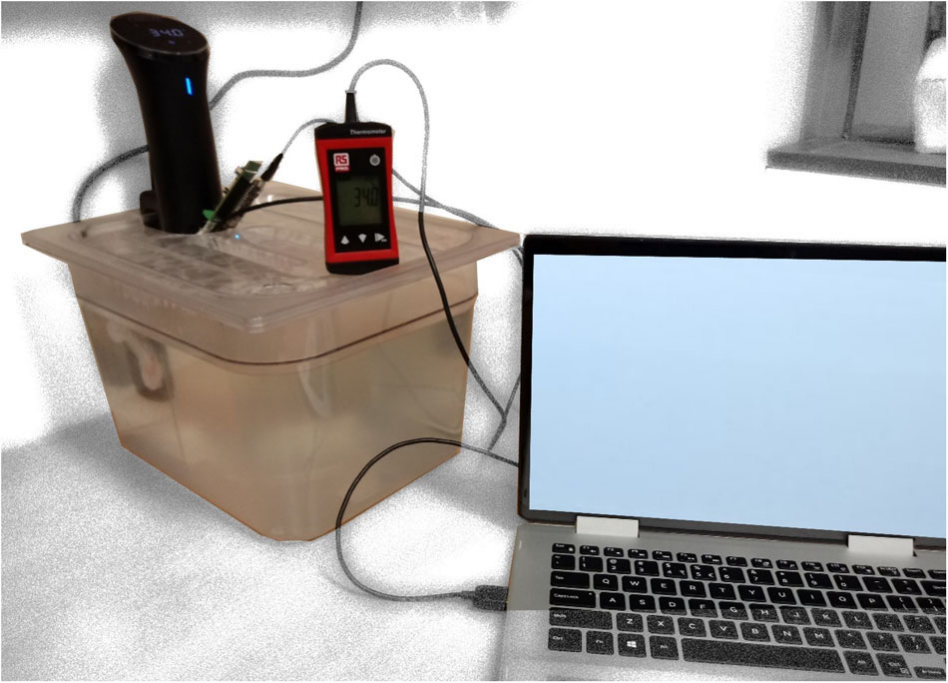
Water bath testing set up showing the precision temperature being set by a heater. The temperature of a 9 l water bath was set to 34.0 °C. An Input Wired Digital Thermometer and the instrumented mouthguard were sampled by the PC GUI program using a MAX30208 Evaluation System. Both the sensor of the Wired Digital Thermometer and the mouthguard are submerged in the water bath.

The in vitro tests are based on the BS EN ISO 80601-2-56:2017 standard, section 201.101.2 for clinical thermometers for body temperature measurement [18]. The tests were undertaken in a well-ventilated room with a mean temperature of 21 °C. Each experiment consisted of an instrumented mouthguard and an RS PRO RS1710 PT1000 Input Wired Digital Thermometer simultaneously submerged into a 9 l circulating water bath that had the temperature set by a heater (Nano, Anova Applied Electronics, USA) with a temperature accuracy of ±0.1 °C. The water bath is set to a specific temperature at least 30 min before the experiment (Fig. 3). Readings of the output of both thermometers were logged every 10 s for 30 min. The water temperature was kept constant throughout each experiment with an accuracy of ±0.1 °C. The water bath was preheated before inserting the devices to ensure that the water was at the desired stable temperature. No effort was made to keep the sensors in the same location or orientation in the water bath throughout the trials, but the sensor were generally placed in close proximity to the heating module.

The BS EN ISO 80601-2-56:2017 section 201.12.1.101 standard determines that a clinical thermometer must have an output temperature range of at least 34–43 °C, with the three temperatures tested being the range: a mid-point of the output range; and within 1 °C of each limit [18]. Therefore, the three temperatures settings used to validate the sensors were 34, 38.5 and 43 °C. These in vitro testing conditions were repeated for each mouthguard that was placed in the water bath. A total of four instrumented mouthguards were tested resulting in four data sets covering the aforementioned three temperatures (34, 38.5 and 43 °C).

### Preliminary in vivo testing

The preliminary in vivo tests collected data from the same instrumented mouthguard that was tested under the in vitro conditions. 3M Tempa DOT Single-Use Clinical Thermometers (with an accuracy of ±0.1 °C) was used as reference measure during in vivo testing. Both thermometers were used to measure the body temperature of one subject for 30 min. At the start and the end of the experiment, the temperature of the buccal side of the right upper first molar (same position of the sensor in the instrumented mouthguard) was measured with the single-use thermometers before the placement of the mouthguard in the mouth. Once the mouthguard was placed, the single-use thermometers were applied every 5 min to measure the sublingual temperature. The mouthguard logged temperature every 10 s. Two mouthguards were tested with the volunteer to obtain the relevant data.

### Data analysis

A basic descriptive analysis was performed. The error results were calculated as the difference between the temperature recorded by the instrumented mouthguard and that of the respective reference sensor. The reference sensors for the in vitro consisted of the Wired Digital Thermometer (RS1710), as well as the temperature sensor in the water bath heater. Results were expressed as mean absolute error, as well as the root mean squared error. Steady-state temperature equilibrium in the mouthguard readings was defined as 60 s of continuous data with a variation below 0.02 °C (the long-term stability error reported for the MAX30208 temperature sensor in the mouthguard). Temperature equilibriums were also confirmed visually by a plateau in temperature readings. For simplicity, statistical analysis of temperature equilibrium was performed on data between 20 and 30 min into the experiment, as it was previously observed in a pilot study that the mouthguard measurements reached temperature equilibrium by then. Readings resulting from spurious events were filtered out using the localized mean and the standard deviation [19]. The data analyses were conducted using Matlab R2019b (Mathworks, Natick, MA, USA).

## Results

### In vitro testing

**Fig. 4:**
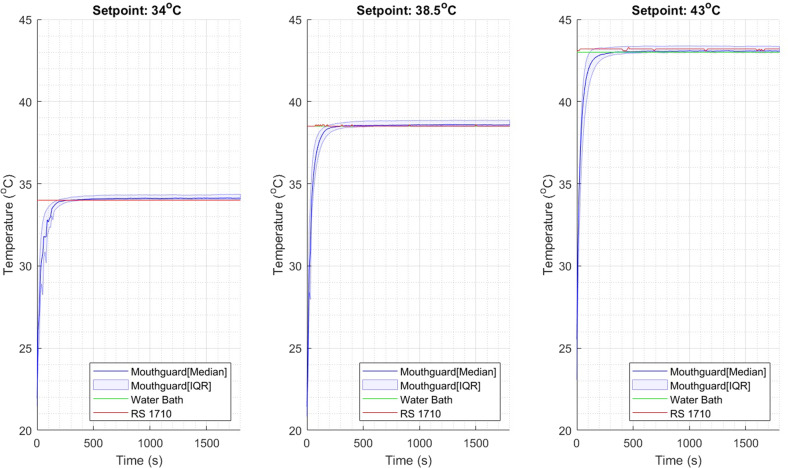
Water bath temperature readings at different setpoints for each temperature sensor. There are two reference sensors (Water bath and RS1710) and one sensor embedded in the mouthguard. All data were synchronized during collection. The IQR represents the interquartile range for the mouthguard data.

A plot of the temperature outputs from the instrumented mouthguard, the RS1710 thermometer and the water bath are shown for the three temperatures in Fig. 4.

The median time for the instrumented mouthguard to reach temperature equilibrium was 380 s (range from 130 to 690 s). For 34 °C the median time was 360 s (range from 130 to 690 s); For 38.5 °C the median time was 425 s (range from 150 to 510 s); For 43 °C the median time was 455 s (range from 170 to 630 s). The mean absolute steady-state error and the root mean squared steady-state error in temperature readings are summarized in Table 1.

**Table 1:** Steady-state error in temperature readings.

Setpoint	Error from RS1710 thermometer mean absolute error ± SD (°C)	Error from RS1710 thermometer root mean squared error ± SD (°C)	Error from water bath thermometer mean absolute error ± SD (°C)	Error from water bath thermometer root mean squared error ± SD (°C)
34 °C	0.13 ± 0.22	0.12 ± 0.25	0.20 ± 0.20	0.20 ± 0.23
38.5 °C	0.18 ± 0.24	0.18 ± 0.27	0.20 ± 0.24	0.19 ± 0.27
43 °C	0.24 ± 0.16	0.24 ± 0.18	0.20 ± 0.23	0.20 ± 0.27
Total	0.21 ± 0.21	0.21 ± 0.28	0.20 ± 0.22	0.20 ± 0.23

Error is calculated by subtracting mouthguard readings from one of the two reference thermometers. The steady-state error combines data points between 1200 and 1800 s from all the experiments at that temperature.

### Preliminary in vivo testing

**Fig. 5:**
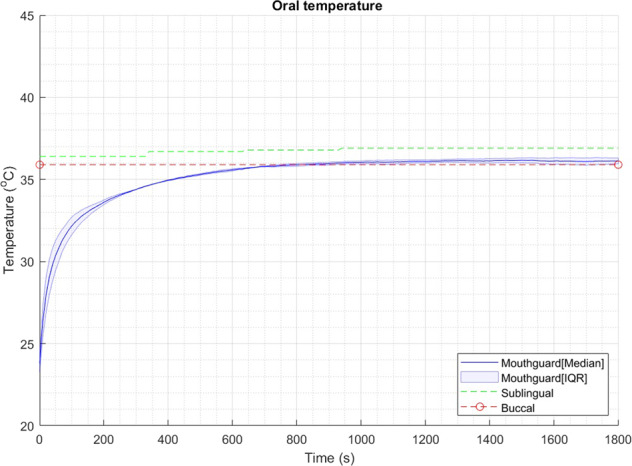
Oral temperature readings. Mouthguard temperature sampled every 10 s, sublingual temperature sampled with single-use thermometers every 5 min, buccal temperature sampled with single-use thermometers before and after placing the mouthguard. The exact time points for buccal measurements are shown as red circles.

A plot of the different temperature outputs against time is shown in Fig. 5. Once inside of the mouth, the median time to steady-state (temperature equilibrium) was 1030 s (ranging from 950 to 1110 s). When compared to the single-use thermometers placed on the same location as the mouthguard sensor, the mean absolute steady-state error (±standard deviation) was 0.23 ± 0.16 °C and the root mean squared error was 0.24 ± 0.21 °C. When comparing with sublingual temperature (representative of body temperature), the instrumented mouthguard had a mean steady-state error of 0.72 ± 0.11 °C and root mean squared error of 0.72 ± 0.15 °C.

## Discussion

This study used a comprehensive approach to assess the steady-state error of temperature sensors embedded in a mouthguard. We compared the data acquired from a semiconductor-based sensor encapsulated in EVA and two reference thermometers. The results show that sensors embedded into a mouthguard can measure temperature with a consistent error across different temperatures. The encapsulation material did not seem to impact the steady-state error of the sensor, similar to the findings from Kirshenblatt et al. that tested the effects of embedding temperature sensors in different materials [20]. However, it is essential to consider the time for reaching steady-state temperature. Minimizing this time will allow for a quicker indication of the user’s temperature. Yet, when hot and cold drinks are consumed the oral temperature can vary between 1 to 71.8 °C [21–23]. These are often transient events that with a fast response rate could yield incorrect temperature interpretation. Increasing the time it takes to reach temperature equilibrium will smooth out the effects of these events.

The water bath test outlined is very comprehensive, but not exhaustive. The error was determined using different set points in the water bath, as this captured the range over which the sensor should operate. This is an essential step, which thus far is not widely applied in the scientific literature. In this study, the mouthguard sensor was envisioned to measure steady-state oral temperature for potential clinical applications. For this reason, the temperature set points tested were based on the human body temperature ranges. Dentistry studies that measure oral temperature during the normal daily routine report an oral temperature varying from 25.9 to 43.5 °C [24]. To accurately measure the temperature in the intra-oral scenarios the temperature sensor should be accessed across the whole range. Although Farella et al. performed water bath tests set at 25, 35 and 45 °C, the purpose of these tests was to validate a pH electrode and not to validate their temperature sensor [25]. To the best of our knowledge, this is the first study that uses a relevant range of temperatures for assessing oral-based temperature sensors that are developed for long-term use. The in vivo tests complement the water bath tests and allow the results to be generalized toward a more real-world application.

The average steady-state error found in both the water bath tests and in the case study is under 0.27 °C, making it suitable for medical applications [18]. Noticeably, even though the temperature sensor used in the mouthguard has a nominal accuracy of ±0.1 °C the overall accuracy of the mouthguard was around ±0.2 °C. Although this result should be considered carefully, due to the small sample size.

Plots of time series data should always be considered for temperature sensors that are developed for continuous monitoring. It was shown that there was a difference between the time it took to reach a steady-state for the water bath and in vivo tests, with the in vivo tests taking longer. This is probably due to airflow and heterogeneous temperature distribution inside of the oral cavity [24]. Better sensor placement and encapsulation could further help to mitigate the effects of these oral temperature gradients. This does also raise some concerns over the protocols currently used to test temperature data loggers designed to monitor the wear time of oral appliances. These sensors are frequently tested in thermostatic water baths assuming the water temperature set at 35 °C as a reliable analog for the oral environment [20, 26, 27]. Our study shows that even if the error from the water bath tests and the oral cavity are comparable, the time components can be very different, making the water bath a limited analog for the oral environment. Indeed, previous studies suggested that water baths tests can’t mimic intra-oral conditions [24]. For the wear-time monitoring application, in which time is the variable of importance, the water bath tests may not be enough to emulate the heterogeneity.

It should be noted that although the fabrication of a wired system allowed for a robust, synchronized data collection strategy, it did require additional effort in terms of waterproofing, limiting the experimentation with more devices and subjects. A wireless design could minimize the risks of water ingression and could be applied in future studies.

Finally, ambient factors such as room temperature were not controlled before placing the temperature sensors into the water bath and could have impacted the time to steady-state. Also, the accuracy of the water bath temperature and the reference sensor is of the same order of magnitude as the mouthguard sensor, limiting a further detailed analysis of the error. This factor, however, should not have a large impact on clinical applications, as errors under 0.1 °C are considered irrelevant [4].

## Conclusions

This study determined the steady-state errors of mouthguards instrumented with temperature sensors using both lab bench and in vivo tests. Across a range of temperatures, the observed steady-state error was appropriate for clinical requirements. However, the time it takes to reach temperature equilibrium in the water bath is significantly lower than that observed in the in vivo experiment, indicating that the water bath may not be a complete representation of the oral environment for applications that need to account for the time components of the readings. This result demonstrated the use of applying a comprehensive process for assessing intra-oral temperature sensors.
